# Usefulness of black boundary artifact on opposed-phase imaging from turbo spin-echo two-point mDixon MRI for delineation of an arthroscopically confirmed small fracture of the lateral talar dome: A case report

**DOI:** 10.1097/MD.0000000000009497

**Published:** 2017-12-29

**Authors:** Eun Hae Park, Kwang-Bok Lee

**Affiliations:** a Department of Radiology; b Department of Orthopedic Surgery, Research Institute of Clinical Medicine of Chonbuk National University—Biomedical Research Institute of Chonbuk National University Hospital, Chonbuk National University Medical School, Korea.

**Keywords:** ankle, black boundary artifact, Dixon MRI, fracture

## Abstract

**Rationale:**

A nondisplaced chip fracture can be missed on MRI. Opposed-phase imaging from mDixon MRI produces an interesting artifact called black boundary artifact. This artifact can provide better contrast at the fracture line resulting in better depiction of a small chip fracture on MRI.

**Patient concerns:**

We present a case of small nondisplaced chip fracture at the lateral talar dome that was well delineated only with the aid of a black boundary artifact after using T2-weighted opposed-phase imaging from turbo spin-echo two-point modified Dixon ankle MRI. In other sequences, the lesion demonstrated a focal full thickness cartilage defect, subtle cortical irregularity, or subcortical bone marrow edema but the full delineation of the fracture line was not possible.

**Diagnoses:**

Opposed-phase imaging from mDixon MRI aided in the diagnosis of lateral talar dome osteochondral fracture (osteochondral lesion), which was confirmed arthroscopically.

**Interventions:**

The osteochondral fragment was removed by arthroscopy.

**Outcomes:**

The patient did well with recovery of full range of motion after 2 months.

**Lessons:**

We have identified a black boundary artifact using opposed-phase imaging from mDixon MRI that can aid in detection of small fracture, which can be missed by conventional MRI, by providing a dark linear signal at the fracture line.

## 1 Introduction

Magnetic resonance imaging (MRI) is unsatisfactory for the depiction of nondisplaced small avulsion fractures or chip fractures because of the low signal intensity of the cortex which cannot be distinguished from a low signal fracture line and partial volume effect.^[1–3]^ However, since ligamentous injury is the most common type of ankle injury, MRI is frequently performed in ankle injury.^[4,5]^

Recently the Dixon technique, described by Dixon^[6]^ in 1984, has been applied in MR imaging of distal extremities, including the ankle. This technique has gained attention because it can achieve uniform fat suppression in multiple joints.^[7,8]^ The opposed-phase imaging provided by the Dixon technique shows a characteristic artifact called an india ink artifact or black boundary artifact, especially at the boundary of different kinds of soft tissue.^[9,10]^

Here we report an arthroscopically confirmed case of a small nondisplaced chip fracture (osteochondral lesion) of the lateral talar dome that was well delineated only with the aid of a black boundary artifact, which was visualized using T2-weighted opposed-phase imaging from turbo spin-echo two-point modified Dixon (mDixon) ankle MRI. In other sequences, the lesion demonstrated a focal full thickness cartilage defect, subtle cortical irregularity, or subcortical bone marrow edema but the full delineation of the fracture line was not possible.

## 2 Case report

A 56-year-old male without any previous medical history presented to our emergency room (ER) with multiple traumas from a 10 meter fall in a construction field. Physical examination revealed a male patient with a body mass index in the normal range and an acutely ill looking appearance. His right lower leg and ankle were swollen and bruised, and he had a 2 cm laceration wound on the plantar aspect of his right foot. The patient's right ankle had limited range of motion due to pain. The patient had tenderness at the right anterolateral aspect of the mid lower leg and anterior aspect of the ankle. There was grade 1 anterolateral instability of the left ankle. The neurologic examination was normal. Based on the patient's clinical history and physical examination, the orthopedic surgeon suspected a fracture of the right fibular diaphysis and ligament injury of the right anterolateral ankle.

**Figure 1:**
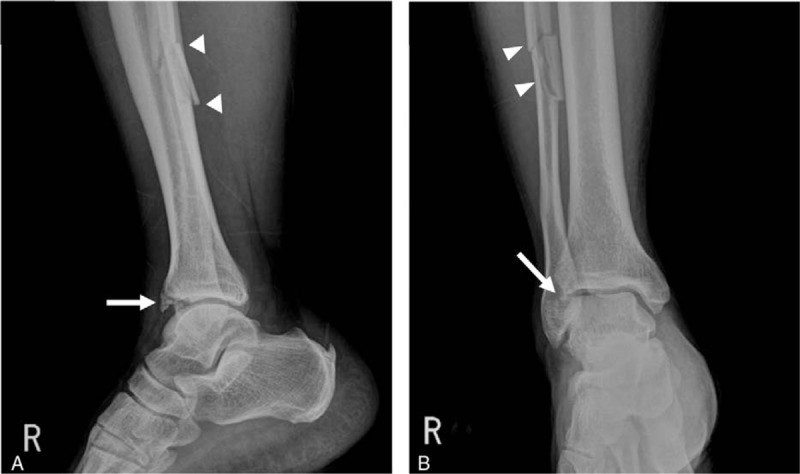
Initial ankle anteroposterior (A) and lateral (B) radiographs demonstrate a segmental fracture of shaft of the right fibula (arrow heads in A and B) and fracture of the anterior lip of the tibial plafond with displacement (arrows in A and B).

Initial radiographs of the ankle in the anteroposterior and lateral views showed fractures at the diaphysis at the fibula and anterior lip of the tibial plafond (Fig. 1). The patient was not able to undergo ankle Mortise view because of his limited range of motion due to extreme pain. In a subsequent lower extremity computed tomography (CT), the orthopedic surgeon in the ER noticed a segmental fracture of the right fibular shaft and the anterior lip of the tibial plafond.

**Figure 2:**
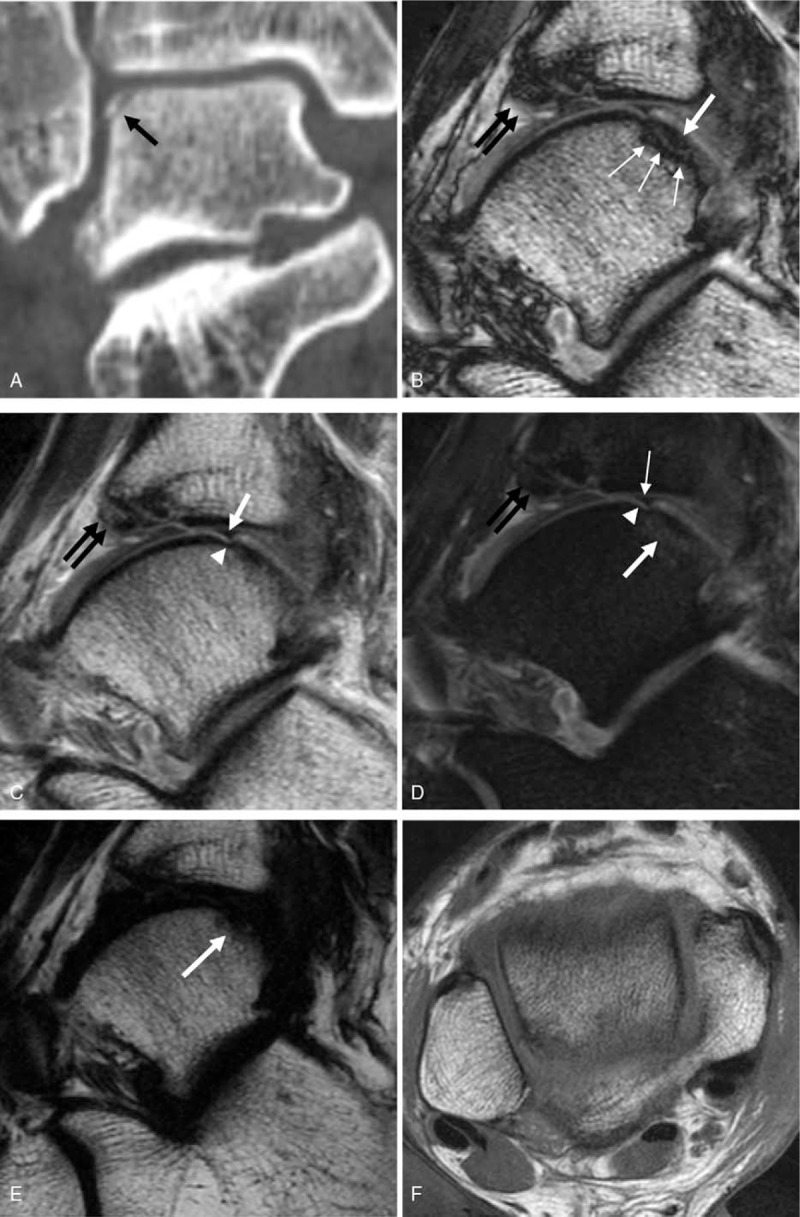
In lower leg CT (A), the chip fracture of the lateral talar dome is well depicted (arrow). There is no significant displacement of the fracture fragment. (B) Sagittal opposed-phase imaging from T2-weighted mDixon imaging demonstrates a wafer-shaped chip fracture at the corresponding area noted from the CT (white thick arrow). The fracture line is clearly delineated by a linear thick dark line caused by black boundary artifact (white thin arrows). (C) Sagittal in-phase imaging from T2-weighted mDixon technique shows a focal wedge-shaped cartilage defect at the talar dome (white arrow). There is subtle cortical irregularity at subjacent cortex (arrowhead). The fracture fragment is not delineated. (D) Sagittal water-only sequence from T2-weighted mDixon imaging shows a fuzzy linear bone marrow edema (white thick arrow). The cartilage defect (white thin arrow) and cortical irregularity (arrow head) are noted. (E) Sagittal fat-only imaging from T2-weighted mDixon imaging demonstrates a focal dark signal alteration at the subcortical area (arrow) which cannot be distinguished from the adjacent cortex or physiologic effusion. (F) In axial T1-weighted image, the fracture line is not clearly depicted. Note the displaced fracture fragment from the anterior tibial plafond is relatively clearly noted in B–D (black double arrows).

To evaluate the ankle ligaments, a turbo spin-echo (TSE) two-point mDixon technique applied to an ankle MRI (Table 1) was performed after procuring written informed consent. In addition to the fractures of the right fibular shaft and tibial plafond, this MRI demonstrated a tiny chip fracture of the lateral talar dome. A tiny wafer-shaped talar dome chip fracture fragment about 7 (anterior–posterior diameter) × 3 (head to toe diameter) mm was clearly delineated only in the sagittal T2-weighted mDixon opposed-phase MRI (Fig. 2B). In T2-weighted mDixon in-phase imaging, which is considered a conventional T2-weighted image, there was a definite focal wedge-shaped cartilage defect at the corresponding area. However, there was only focal and subtle cortical irregularity and the cortical step-off was not definite (Fig. 2C). In a T2-weighted mDixon water-only image, which is considered a conventional fat-suppressed T2-weighted imaging, the cartilage lesion and focal cortical irregularity were once again noted, and the subcortical bone marrow edema was additionally confirmed. In these 2 sequences, a fracture was suspected, but the radiologists could not fully delineate the fracture line (Fig. 2D). In T2-weighted mDixon fat-only imaging, there were dark signal alterations at the subcortical region, but these were not considered fractures (Fig. 2E). T1-weighted imaging was obtained in the axial plane, and the fracture line was not depicted in this plane (Fig. 2F). In a CT image reviewed by an experienced musculoskeletal radiologist, there was a lateral talar shoulder cortical fracture at the identical area where the chip fracture was noted (Fig. 2A) from the T2-weighted mDixon opposed-phase image. In addition, there was a grade 2 injury to the anterior talofibular ligament with severe subcutaneous swelling of the ankle.

**Table 1:**
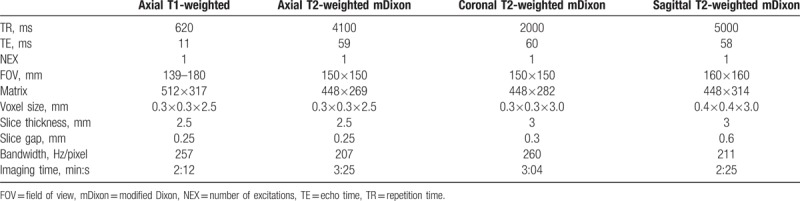
Parameters of magnetic resonance imaging.

**Figure 3:**
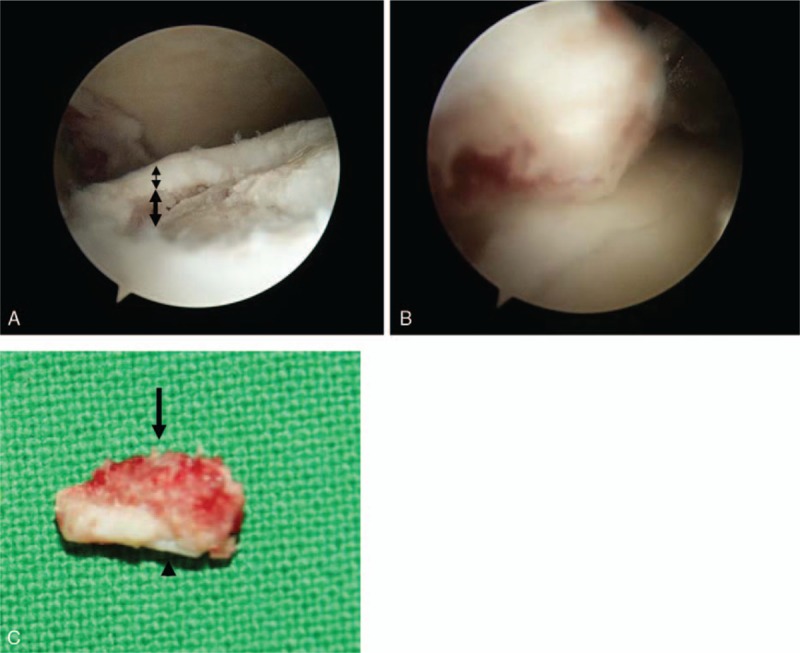
On arthroscopy, there is an osteo (thick double-headed arrow)-chondral (thin double-headed arrow) defect from the chip fracture at the posterolateral talar dome (A). The fracture fragment was removed by basket (B). The bony part is located in the superior region (arrow) and the relatively smooth cartilage portion (arrowhead) is in the inferior region (C).

During ankle arthroscopy, there was a free floating osteochondral fragment about 4 x 8 mm at the posterolateral talar shoulder, which was removed with basket forceps (Fig. 3), and microfractures were performed at the posterolateral talar cortical fracture site.

The patient did well after the arthroscopy with recovery of full range of motion after 2 months.

## 3 Discussion

In the present case, a small chip fracture of the lateral talar dome was only fully delineated in the opposed-phase imaging from T2-weighted mDixon MRI. In the other three sequences (T2-weighted mDixon in-phase, water-only, and fat-only sequence) the fracture was missed or only suspected. The fracture line was not definite and the fracture fragment could not be delineated.

MRI is unsatisfactory for the depiction of small avulsion fractures or chip fractures. Because of the low signal intensity of the cortex, thin cortical avulsion may be interpreted as ligament avulsion.^[3,11]^ According to Palmer et al^[2]^, cases of nondisplaced fracture without adjacent bone marrow edema may be difficult to detect. There was a previous report of MRI findings in 12 patients with Segond fractures that were only visible one third of the time because edema and hemorrhage in the surrounding soft tissue obscured the small fractures.^[1]^

The Dixon imaging technique first described by Dixon,^[6]^ is an alternative method for fat suppression based on the chemical shift phenomenon. Until recently, this technique was difficult to implement in MRI of joints mainly due to greater inhomogeneity of the magnetic field. However, advanced techniques and substantial improvements have enabled successful application of this technique in clinical joint imaging including peripheral joints such as the ankle.^[7,8]^

A single acquisition of mDixon imaging provides 4 image sets. First, in-phase and opposed-phase images are obtained, and from pixel-by-pixel postprocessing of the data, additional water-only and fat-only images are calculated.^[12,13]^

In opposed-phase images, the interface between water and fat is demonstrated by a thick dark line since the voxels there contain both water and fat; the signals from each tissue cancel each other out resulting in a completely nulled signal. This is a characteristic artifact so-called an india ink artifact or black boundary artifact.^[9,10]^ On abdominal imaging, margins of the organs appear as if they are outlined with black ink. In the present case, this interesting artifact provided black ink like line at the fracture site, and thus the lateral talar dome fracture line was well delineated in the opposed-phase image. This is because, technically, the boundary of the fracture line is where the water is present within the mainly fat-containing bone marrow.

In the present case, the fracture site in the T2-weighted mDixon in-phase image (similar to a conventional T2-weighted image) demonstrated only focal and subtle cortical irregularity. Cortical step-off was not definite, but given that there was a full thickness cartilage defect at the same site, a fracture (osteochondral lesion) was suspected.

In addition, T2-weighted mDixon water-only imaging revealed a fuzzy wafer-shaped focal linear bone marrow edema at the fracture site. The nature of T2-weighted Dixon water-only image is similar to that of fat-suppressed T2-weighted image including short tau inversion recovery (STIR): hyperintense edema to the hypointense bone marrow.^[14]^ On comparison to the CT image, we believe this fuzzy linear edema indicated the location of the fracture line. However, the fracture line still could not be distinguished from a bone marrow edema caused by contusion.

In the fat-only sequence, the fracture site appeared as a low signal but could not be distinguished from the low signal cortex or physiologic joint effusion. Although a systemic comparison of fat-only sequences and other sequences has not been performed, Wohlgemuth et al^[14]^ mentioned that the fat-only sequence better depicted the fracture line compared with water-only or in-phase sequences. This is explained by the similarity of fat-only imaging to T1-weighted image in which normal yellow bone marrow, edema, and the fracture line are hypointense. However, the fracture in their case was not a chip fracture (spiral shaft fracture of the proximal phalanx) and definite cortical disruption was also noted in other sequences. In our case, the fracture fragment was tiny (diameter about 0.4 cm), and hence, the bone marrow of the fragment was probably not fully demonstrated in fat-only imaging because of the partial volume effect.

Usually in MRI, fractures are known to be best visualized with T1-weighted image, and they appear as a low signal intramedullary line extending to the inner cortical margin.^[15,16]^ However, the T1-weighted image in this patient was obtained in the axial plane, and the fracture line was not depicted in this plane because of the partial volume effect (Fig. 2F).

While a transchondral fracture (osteochondral lesion) of the medial talar dome is not as strongly associated with trauma, a transchondral fracture of the lateral talar dome is almost always related to a history of antecedent trauma.^[17]^ The most common mechanism is a strong inversion force with dorsiflexion of the foot that affects the mid-to-anterior aspect of the talar dome. However, we believe that our patient might have fallen with his foot in dorsiflexion resulting in an anterior tibial lip fracture and posterior lateral talar dome osteochondral lesion.

In summary, the black boundary artifact on mDixon opposed-phase imaging may play an important role in identifying a fracture with MRI, especially for a small nondisplaced chip fracture.
